# Anti-lymphoma Activity of Acyclic Terpenoids and Its Structure–Activity Relationship: In Vivo, In Vitro, and In Silico Studies

**DOI:** 10.3390/ijms26125683

**Published:** 2025-06-13

**Authors:** Fernando Calzada, Jesica Ramírez-Santos, Rosa María Ordoñez-Razo, Miguel Valdes, Claudia Velázquez, Elizabeth Barbosa

**Affiliations:** 1 Unidad de Investigación Médica en Farmacología, Unidad Médica de Alta Especialidad, Hospital de Especialidades, Centro Médico Nacional Siglo XXI, Instituto Mexicano del Seguro Social, Mexico City CP 06720, Mexico; valdesguevaramiguel@gmail.com; 2 Unidad de Investigación Médica en Genética Humana, Unidad Médica de Alta Especialidad, Hospital de Pediatría, Centro Médico Nacional Siglo XXI, Instituto Mexicano del Seguro Social, Mexico City CP 06725, Mexico; romaorr@yahoo.com.mx; 3 Instituto Politécnico Nacional, Sección de Estudios de Posgrado e Investigación, Escuela Superior de Medicina, Mexico City CP 11340, Mexico; rebc78@yahoo.com.mx; 4 Área Académica de Farmacia, Instituto de Ciencias de la Salud, Universidad Autonoma del Estado de Hidalgo, San Agustin Tlaxiaca CP 42076, Mexico; cvg09@yahoo.com

**Keywords:** anti-lymphoma activity, U-937 cells, molecular docking, acyclic terpenoids, Bcl-2, DHFR, HMG-CoA reductase, FASN

## Abstract

Terpenoids are a large group of molecules present in several plant species and in many essential oils reported with cytotoxic and anticancer properties. The aim of this study was to evaluate the anticancer activity of eleven acyclic terpenes; seven monoterpenoids: geranyl acetate (C1), geranic acid (C2), citral (C3, mixture of neral and geranial), geraniol (C4), methyl geranate (C5), nerol (C6) and citronellic acid (C7); two sesquiterpenes: farnesal (C8) and farnesol (C9); and one triterpene: squalene (C10), using in vivo, in vitro, and in silico models. Anti-lymphoma activity was evaluated using male Balb/c mice inoculated with U-937 cells. Cytotoxic activity was evaluated using the WST-1 method. Computer tools were used to obtain a molecular docking study, measuring pharmacokinetic and toxicological properties of the acyclic terpenoids with greater antitumor activity. The results showed that the terpenoids with the highest cytotoxic and nodal growth inhibitory activity were C3, C4, C6, and C9, and their effects were better compared to MTX. The data obtained suggest that the anti-lymphoma activity could be due to the presence of the aldehyde, hydroxyl, and acetate groups in the C1 of the monoterpenes and sesquiterpenes evaluated. The theoretical results obtained from molecular docking showed that geranial (C3A), neral (C3B), C9, and C6 terpenoids obtained a higher affinity for the HMG-CoA reductase enzyme and suggest that it could be a target to induce anti-lymphoma activity of bioactive terpenoids. Our study provides evidence that C3, C6, and C9 could be potential anticancer agents for the treatment of histiocytic lymphoma.

## 1. Introduction

Non-Hodgkin’s lymphomas (NHLs) are a group of neoplasms that affect lymphoid organs, mainly affecting the lymph nodes. They are generally classified as indolent and aggressive lymphomas [1]. NHL continues to be a challenge due to its different subtypes, as each type has different etiological, immunophenotypically, genetic characteristics, and response to treatment [2]. NHL has been associated with several key factors: it has been observed that it is increasing in people with advanced ages and in the population with young ages in developing countries; abnormalities of the immune system; hepatitis C, Epstein–Barr virus, and human immunodeficiency virus (HIV) infections; environmental causes; and patient status (obesity, immunological abnormalities, and chronic inflammation) [3].

In Mexico, NHL is the sixth most common cancer and the types with the highest incidence are diffuse B-cell lymphoma and follicular lymphomas [4].

Some of the aggressive lymphomas, such as diffuse B-cell lymphoma (diffuse B-cell lymphoma) and high-grade B-cell lymphoma, can be treated with curative intent using combination chemotherapy regimens [5]. On the other hand, indolent lymphomas, such as follicular lymphoma, are considered incurable in advanced stages [6]. Chemotherapy treatments are used based on the stage, age, and histologic type of the tumor, usually including the regimen in combination using cyclophosphamide, hydroxydaunorubicin, vincristine, and prednisone (CHOP). As standard, CHOP plus rituximab is used to treat one of the most common lymphomas, diffuse B-cell lymphoma [7]. However, these drugs are non-selective and generate unwanted side effects in the short and long term, causing a decrease in patients’ quality of life [8,9]. Some of the side effects have been reported to be severe infections from the weakened immune system, leukopenia, myelosuppression, liver toxicity, and mucositis [8,9]. Given this context, the search for new antitumor agents with better efficacy and greater selectivity remains a challenge.

One important alternative with anticancer activity which recently has been studied is the terpenoids, which are molecules that make up the broadest group present in most plants. Currently, more than 80,000 terpenoids have been identified as being economically important due to their use in the food, cosmetologically, and pharmacological industries [10]. Terpenoids are the molecules present in several essential oils whose reports have confirmed their medicinal and pharmacological properties. These molecules have been shown to possess pharmacological activities such as anti-allergic, antibacterial, antiparasitic, antioxidant, antifungal, antiviral, and anticancer [11,12]. Anticancer and antitumor activity of monoterpenoids, diterpenoids, sesquiterpenes, and triterpenoids were demonstrated in several cancer cell lines. In addition, terpenoids have been proposed to have high efficacy as multitarget molecules, with some molecules exhibiting low toxicity and being well tolerated by patients in clinical trials [13]. Some of its proposed mechanisms of action play a role in the induction of apoptosis through caspase activation, initiation, and execution; deoxyribonucleic acid (DNA) damage; mitochondrial damage; reactive oxygen species (ROS) generation; cell cycle arrest; topoisomerase I and II suppression; downregulation of nuclear factor of activated T cells 2 (NFAT-2); factor nuclear kappa B (NF-Kb); and reduction of B-cell lymphoma 2 (Bcl-2) expression [12,13]. Currently, some terpenoids are in various phases of clinical trials, such as menthol in breast, colon, esophagus, gastric, and cervical cancer [14,15,16]; D-limonene in breast cancer and pancreatic cancer [17]; and β-elemen against non-small cell lung cancer [18]. All these reports confirm that terpenoids could be antitumor agents, and that more studies are needed to support their pharmacological activities.

One of the most therapeutically important targets in NHLs is Bcl-2; overexpression of this protein is common in many NHL subtypes [19]. The dysregulation of apoptosis is due in part to the overexpression of Bcl-2 in cancer cells. This premise has led to continued research and the obtaining of potential Bcl-2 inhibitor molecules for the treatment of hematological cancers [19,20,21]. Other promising agents for cancer are folate metabolism inhibitors, drugs that have shown efficacy on several types of cancer including NHLs; for example, methotrexate (MTX) is a widely used drug to treat NHL and is an inhibitor of the enzyme dihydrofolate reductase (DHFR), resulting in the reduction of THF and slowing down of DNA synthesis and cell proliferation [22,23].

On the other hand, recent studies have shown that altered fatty acid metabolism is an important oncogenic factor in several types of cancer including NHLs [24]. Fatty acid synthase (FASN) is the key enzyme in fatty acid synthesis, and in recent decades the role it plays in tumor initiation, growth, cancer cell survival, metastasis, and therapeutic resistance has been reported [25,26]. Another enzyme that has gained importance in recent years is 3-hydroxy-3-methylglutaryl-CoA reductase (HMG-CoA reductase), which regulates the mevalonate pathway. This pathway is implicated in cancer progression through the prenylation of proteins such as rat sarcoma virus (RAS), Ras-homologous (Rho), and guanosine triphosphate-hydrolyzing proteins (GTPases) relevant to several types of cancers related to cell growth and proliferation [27,28,29].

**Figure 1:**
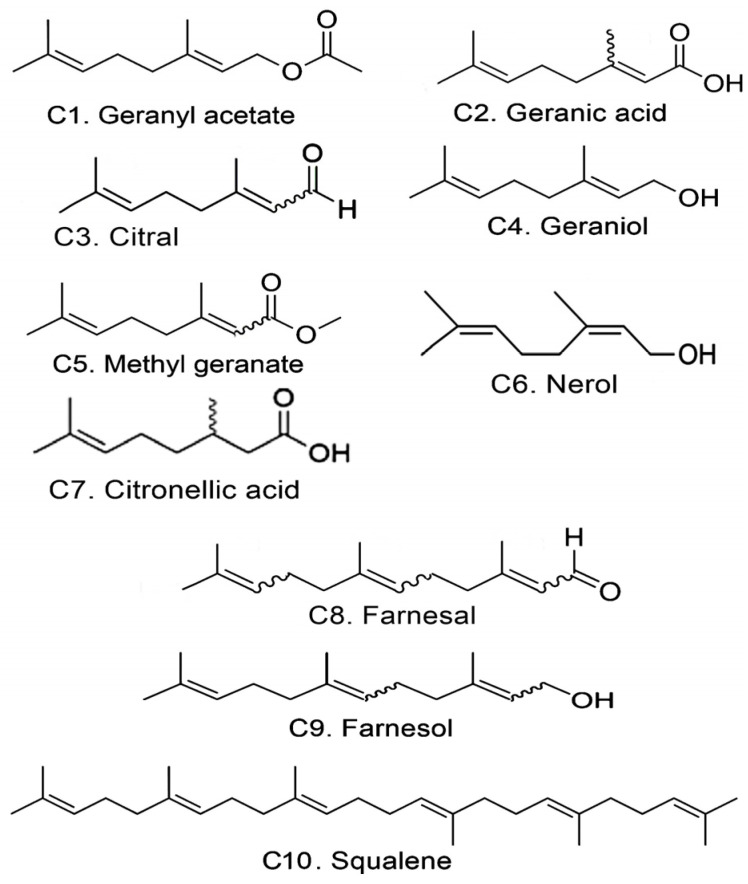
Structure of acyclic terpenoids proposed for the study.

In previous studies, the anti-lymphoma activity and cytotoxic properties of three acyclic terpenoids, farnesyl acetate, geranylgeraniol, and phytol, were evaluated against the U-937 cell line, obtaining promising results [30,31]. Taking into account our history with this type of molecules, we propose to carry out a new study on the anti-lymphomatic activity of other molecules with an acyclic terpenoid structure (Figure 1). Therefore, the objective was to determine the anti-lymphoma activity and cytotoxic properties of seven monoterpenoids: geranyl acetate (*E*-3,7-Dimethyl-2,6-octadien-1-yl acetate), geranic acid (*Z* and *E* diastereoisomers), citral (*Z* and *E* diastereoisomers), geraniol (*E*-3,7-Dimethyl-2,6-octadien-1-ol), methyl geranate (*Z* and *E* diastereoisomers), nerol (*Z*-3,7-Dimethyl-2,6-octadien-1-ol), and citronellic acid (*R* and *S* enantiomers) (C1–C7 respectively); two sesquiterpenes: farnesal and farnesol (*Z* and *E* diastereoisomers) (C8–C9 respectively); and a triterpene: squalene (C10) by in vivo and in vitro tests to determine its pharmacological activity. In addition, in silico assays were used to explore its toxicological and pharmacokinetic properties, and molecular coupling studies were carried out to determine its probable binding activity to the enzymes Bcl-2, DHFR, HMG-CoA reductase, and FASN.

## 2. Results

### 2.1. Cytotoxic Activity

**Figure 2:**
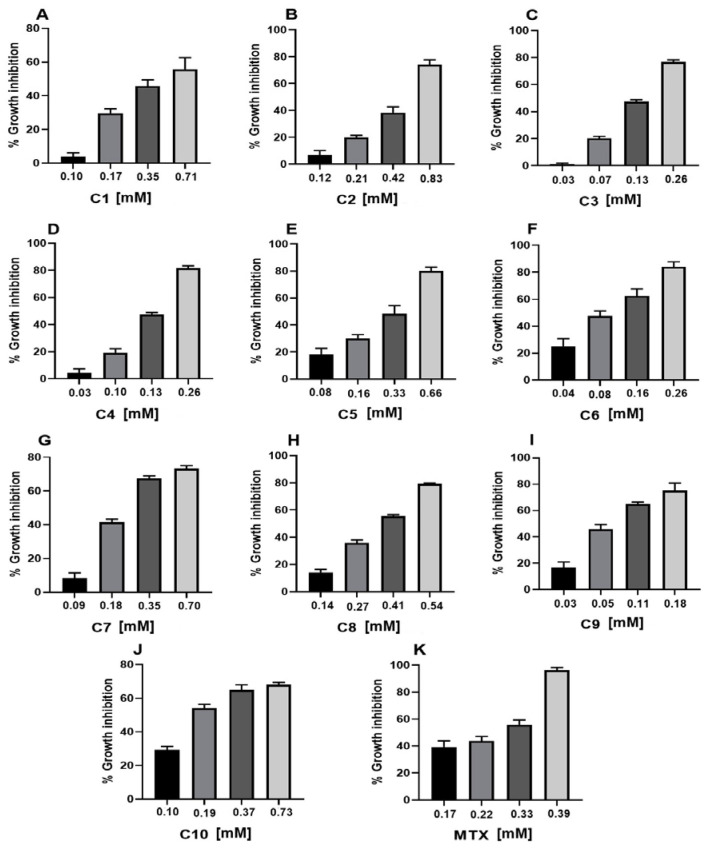
Cytotoxic activity of acyclic terpenoids in U-937 cell line. Representative graphs show inhibition of cell growth caused by geranyl acetate (**A**), geranic acid (**B**), citral (**C**), geraniol (**D**), methyl geranate (**E**), nerol (**F**), citronellic acid (**G**), farnesal (**H**), farnesol (**I**), squalene (**J**), and MTX (**K**) at different concentrations after 24 h of exposure, (n = 3).

The cytotoxicity results against the U-937 cell line of all acyclic terpenoids and MTX were dose-dependent (Table 1 and Figure 2). In addition, the terpenoids farnesol, nerol, citral, and geraniol evaluated obtained a better and lower CC_50_ compared to the reference drug MTX (0.25 ± 0.01 mM). The terpenoids that obtained a lower CC_50_ were farnesol (CC_50_ 0.09 ± 0.01 mM) and nerol (CC_50_ 0.11 ± 0.01 mM). The terpenoids obtained with a CC_50_ higher than MTX were C1, C2, C5, C7, and C8. In addition, the compound that obtained a cytotoxic effect comparable to MTX was C10 (CC_50_ 0.26 ± 0.01 mM).

### 2.2. Anti-lymphoma Activity

**Figure 3:**
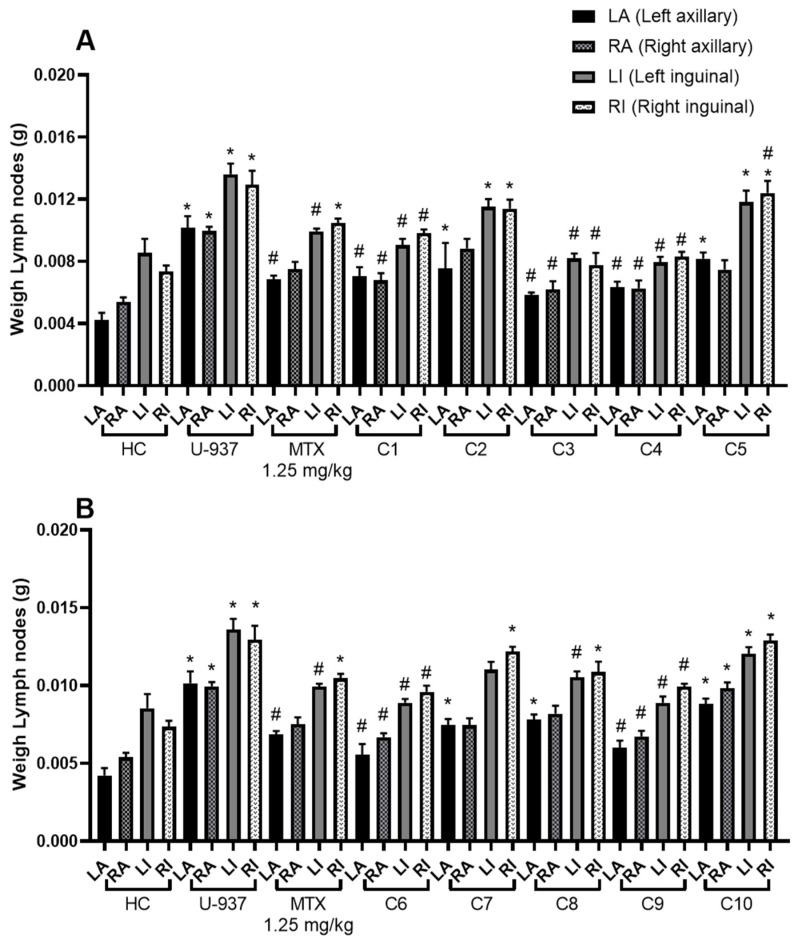
Lymph node weight (g) of male mice. The graph shows the weight of the axillary and inguinal lymph nodes as follows: left axillary (LA), right axillary (RA), left inguinal (LI), and right inguinal (RI). Healthy control (HC tween 80, 2% *v*/*v* in water), untreated control (U-937), methotrexate (MTX) 1.25 mg/kg. (**A**) Geranyl acetate (C1), geranic acid (C2), citral (C3), geraniol (C4), methyl geranate (C5); (**B**) nerol (C6), citronellic acid (C7), farnesal (C8), farnesol (C9), and squalene (C10) at 10 mg/kg. Results obtained by one-way ANOVA analysis followed by Dunnett’s test for multiple comparison. The data are expressed as mean ± SEM, (*n* = 6); * *p* < 0.05 vs. HC, # *p* < 0.05 vs. U-937.

To obtain the results of the anti-lymphoma activity of acyclic terpenoids at the dose of 10 mg/kg and MTX at 1.25 mg/kg, the weight of the left and right axillary and inguinal lymph nodes of male mice was obtained (Figure 3). In all groups, including the healthy control, the trend of greater weight of the inguinal nodes compared to the axillary nodes was maintained.

The highest percentage of lymph node growth inhibition was obtained by citral (C3) with a percentage of 88.06 ± 3.44%. In addition, monoterpene alcohols that also showed a good effect and a significant difference on nodal growth inhibition compared to MTX were geraniol (84.28 ± 2.27%), nerol (75.20 ± 4.33%), and farnesol (71.37 ± 3.86%) at the dose of 10 mg/kg. The effect of terpenoids C1, C7, and C8 was comparable to MTX (56.17 ± 3.11). The compounds that obtained the least effect were C10 and C2 (Table 2).

### 2.3. Toxicoinformatic and Pharmaceutical Analysis of Acyclic Terpenoids

Considering the in vitro and in vivo results, the physicochemical, pharmacokinetic, and toxicological properties of the terpenoids C3, C9, and C6 were predicted (Table 3). In addition, geranial (C3A) and neral (C3B) were included in the evaluations, considering that citral is composed of the mixture of these two terpenoids. The predictive values of terpenoids showed that C3A, C3B, C9, and C6 meet the physicochemical criteria of the Lipinski, Veber, and Egan rule; for Ghose conditions, compounds C3A, C3B, and C6 did not meet the molecular weight (MW) criterion as they had a MW < 160. In general, the pharmacokinetic parameters of all the terpenoids analyzed showed optimal and accepted values. All terpenoids obtained a high human intestinal absorption value, are permeable to the blood–brain barrier, and the value of their distribution volume was optimal. However, plasma protein binding was not optimal for all terpenoids, since the values obtained were > 90%. In addition, terpenoids C3A, C3B, and C9 obtained the probability of being inhibitors or substrates of any of the isoenzymes that make up cytochrome P450. C9 obtained the probability of being a cytochrome P450 1A2 (CYP1A2) inhibitor, and in the case of C3A and C3B, it was found that they are possible inhibitors and substrate of cytochrome P450 2C19 (CYP2C19). In addition, the predictive results of toxicity suggest that the acyclic terpenoids tested are non-mutagenic, carcinogenic, and neurotoxic. As for hepatotoxicity in humans, they indicated terpenoids have a low probability of causing it. In addition, the toxicological classification of terpenoids C3A, C9, C6, and C4 indicated that they belonged to class V and they can be harmful if ingested; furthermore, C3B was classified in category 4, that is, harmful if ingested.

### 2.4. Molecular Docking Studies of Acyclic Terpenoids

**Figure 4:**
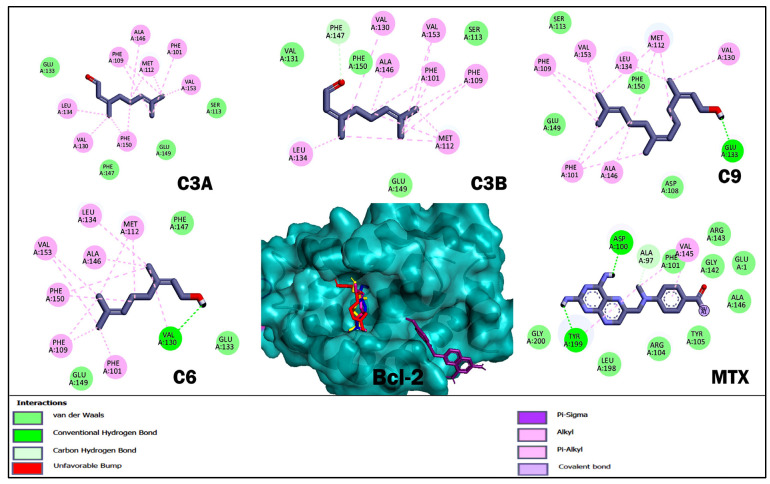
Results of molecular docking of the enzyme Bcl-2 and terpenoids. The images show the position of the binding site and the 3D and 2D interactions. In the protein complex, the colors of terpenoids are shown as follows: geranial (C3A) magenta, neral (C3B) blue, nerol (C6) yellow, farnesol (C9) red, and methotrexate (MTX) in purple. In the 2D diagram, polar-type interactions are marked in green (van der waals, conventional hydrogen bond or carbon hydrogen bond); and non-polar-type interactions are marked in pink (alkyl or pi-alkyl), lilac (covalent bond), and purple (pi-sigma).

**Figure 5:**
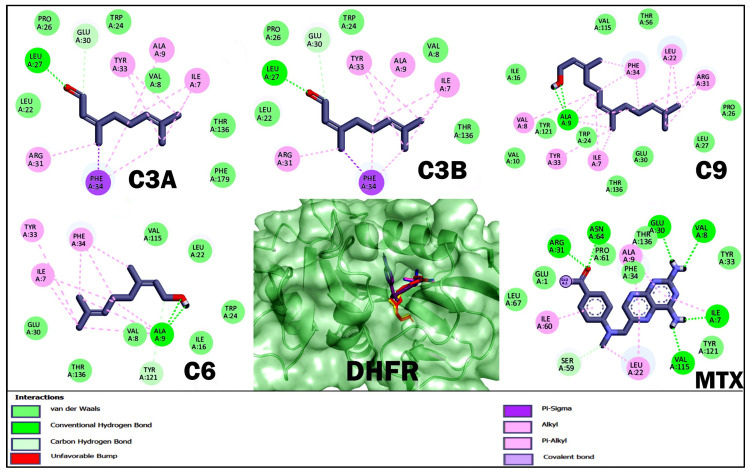
Results of molecular docking of the DHFR enzyme and terpenoids. The images show the position of the binding site and the 3D and 2D interactions. In the protein complex, the colors of terpenoids are shown as follows: geranial (C3A) magenta, neral (C3B) blue, nerol (C6) yellow, farnesol (C9) red, and methotrexate (MTX) in purple. In the 2D diagram, polar-type interactions are marked in green (van der waals, conventional hydrogen bond or carbon hydrogen bond); and non-polar-type interactions are marked in pink (alkyl or pi-alkyl), lilac (covalent bond), and purple (pi-sigma).

**Figure 6:**
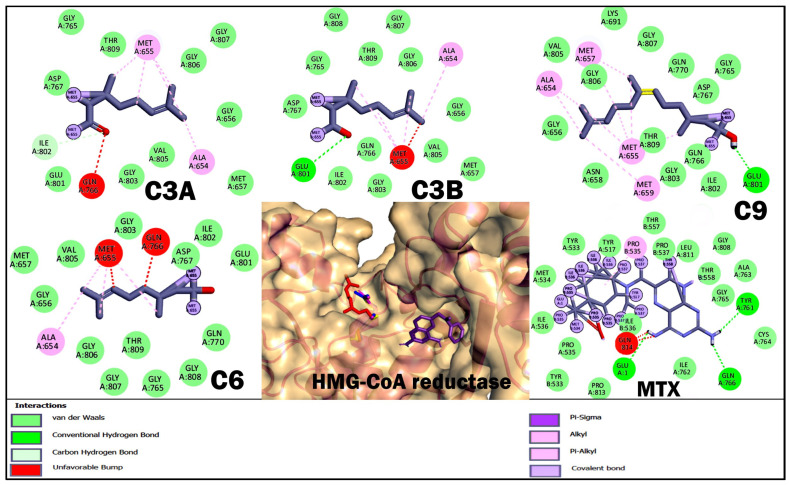
Results of molecular docking of the enzyme HMG-CoA reductase and terpenoids. The images show the position of the binding site and the 3D and 2D interactions. In the protein complex, the colors of terpenoids are shown as follows: geranial (C3A) magenta, neral (C3B) blue, nerol (C6) yellow, farnesol (C9) red, and methotrexate (MTX) in purple. In the 2D diagram, polar-type interactions are marked in green (van der waals, conventional hydrogen bond or carbon hydrogen bond); and non-polar-type interactions are marked in pink (alkyl or pi-alkyl), lilac (covalent bond), purple (pi-sigma) and red (unfavorable bump).

**Figure 7:**
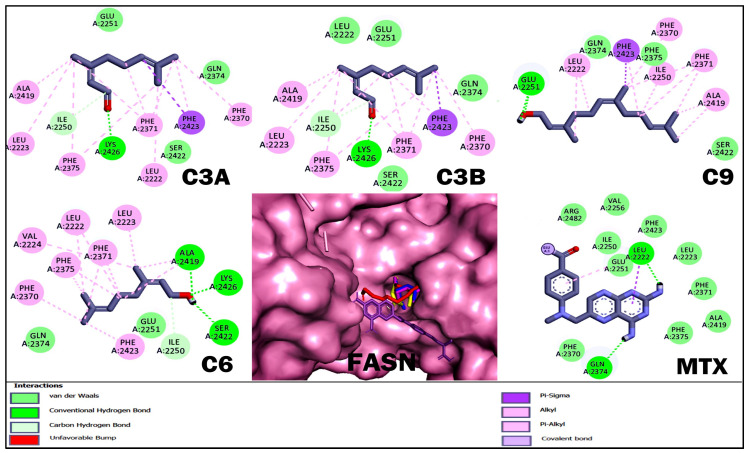
Results of the molecular docking of the FASN enzyme and terpenoids. The images show the position of the binding site and the 3D and 2D interactions. In the protein complex, the colors of terpenoids are shown as follows: geranial (C3A) magenta, neral (C3B) blue, nerol (C6) yellow, farnesol (C9) red, and methotrexate (MTX) in purple. In the 2D diagram, polar-type interactions are marked in green (van der waals, conventional hydrogen bond or carbon hydrogen bond); and non-polar-type interactions are marked in pink (alkyl or pi-alkyl), lilac (covalent bond), and purple (pi-sigma).

With respect to molecular docking studies, we used Bcl-2, DHFR, HMG-CoA reductase, and FASN as targets and the three terpenoids with the best cytotoxic and anti-lymphoma activity obtained in in vitro and in vivo studies as ligands (Table 4, Figure 4, Figure 5, Figure 6 and Figure 7). For the selection of targets, Bcl-2 was considered because it is widely used to obtain possible inhibitors used in the clinic in hematological cancers, including NHLs [20,21]. The DHFR enzyme was selected because it was the target of MTX, the control drug used in our study and used as a treatment for NHL [23,32]. The enzyme HMG-CoA reductase and FASN were selected by obtaining high credibility and a high score in the PASS program for the prediction of possible targets and biological activity of the terpenoids C3A, C3B, C9, and C6. The results for Bcl-2 showed that MTX and C9 obtained the best binding energy compared to the other terpenoids with a ΔG −5.8 and −5.76 kcal/mol, respectively. Terpenoids obtained a greater number of non-polar interactions and joined with some amino acids important for the anti-apoptotic activity of Bcl-2 such as Phe 101, Phe 109, Met 112, and Ala 146. In the results for the DHFR enzyme, MTX obtained the best binding energy with a ΔG −8.58 kcal/mol. The terpenoids interacted with several binding residues described for the hDHFR substrate such as Ile 7, Leu 22, Arg 31, and Glu 30; in addition, C9 obtained two polar-type interactions at Val 115 and Tyr 121 key residues from the DHFR catalytic site and which it shared with MTX. Regarding the data obtained for the enzyme HMG-CoA reductase, C9 obtained the best binding energy with a ΔG −6.72 kcal/mol; in addition, all terpenoids obtained a polar-like interaction in the key residue Asp 767 of the enzyme’s active site and shared interactions with residues of the catalytic portion of the enzyme. On the other hand, the results for the FASN enzyme showed that C9 obtained a better affinity with a ΔG −5.79 kcal/mol. However, the terpenoids did not bind to any amino acids in the triad of the catalytic site described.

In general, the terpenoids evaluated obtained a better affinity with the enzyme HMG-CoA reductase; in addition, C9 was the one that obtained the best affinity in all the targets analyzed compared to the other terpenoids.

## 3. Discussion

In the cytotoxicity test, terpenoids were evaluated against the U-937 cell line, which corresponds to a highly aggressive histiocytic non-Hodgkin lymphoma (Table 1) [33]. The terpenoids C9, C6, C3, and C4 showed better cytotoxic activity against U-937 cells compared to MTX, a drug widely used worldwide. In addition, the terpenoids that obtained a better cytotoxic effect were monoterpene alcohols with the hydroxyl group in C1, farnesol (C9) (0.09 ± 0.01 mM), nerol C6 (CC_50_ 0.11 ± 0.01 mM), and geraniol C4 (CC_50_ 0.17 ± 0.01 mM); and citral (a mixture of geranial and neral) with the aldehyde unsaturated in C1 and C3 (CC_50_ 0.16 ± 0.01 mM).

Once CC_50_ was obtained, we decided to evaluate terpenoids in male mice with induced lymphoma (Figure 3 and Table 2). It is important to note that the dose of 10 mg/kg body weight used in the animals was established in accordance with our previous studies, observing that at that dose some acyclic terpenoids reached a higher percentage of nodal growth inhibition. In addition, the dose established for MTX was 1.25 mg/kg body weight because in previous studies it was obtained as a safe dose to work experimentally in the model at repeated doses, since some female and male mice of the Balb/c strain died when this dose was increased [33,34]. In relation to the structure–activity study, few changes were observed; however, we can mention the following: the groups treated with the monoterpenes C3, C4, and C6 obtained a good effect and the highest percentage of inhibition of lymph node growth compared to the other terpenoids and MTX. The better inhibitory activity could be due to the presence of the aldehyde group in C1 (for C3, an isomeric mixture of geranial and neral) and then to the hydroxyl group. In addition, when comparing C4 and C6 terpenoids, it was observed that the trans-to-cis configuration generated a slight change in activity. When analyzing the effect of C3, C8, and C9, we realized that the addition of an isoprene decreases biological activity; in addition, C10 with three isoprene units and without oxygenated groups completely lost its inhibitory activity on lymph node growth. The comparison between C2 and C1 showed that by blocking the functionality of carboxylic acid by an ester, biological activity improves substantially. In addition, we realized that an ester group in the C1 of the monoterpene structure, as is the case of C1, obtains a better effect compared to an ether as in the case of C5. On the other hand, when comparing C2 and C7, it was observed that the reduction of the double bond α and β did not generate significant changes in biological activity. It is important to mention that the results obtained in the present study present an uncertainty factor of isomeric mixing or racemic mixing. For example, the compounds with the highest anti-lymphoma activity would be the case of C3, which is a mixture of the terpenoids geranial and neral, and C9, which is an isomeric mixture.

On the other hand, some studies support our findings on cytotoxic and antitumor activity. C4 and C1 have been shown to have cytotoxic activity against the colon cancer cell line (colo-205), with C4 having better activity compared to C1 [35]. Other reports have shown that terpenoids C3, C4, and C6 obtained an IC_50_ in the range of 24 μg/mL and 34 μg/mL and induced apoptosis in breast adenocarcinoma cancer (MCF-7) and bladder cancer (T24) cell lines by increasing ROS, loss of mitochondrial membrane potential, and activation of caspases 7 and 9. In addition, the authors observed that upregulation of proteins such as Bcl-2-associated X protein (Bax) and Bcl-2-associated activating factor 1 (Bak) and downregulation of Bcl-2, RNA-activated ER-like protein phosphorylated kinase (pERK), and phosphorylated protein kinase B (pAkt) were obtained [36,37]. For C3, a study revealed that it induced autophagy and apoptosis in head (SCC15) and neck (CAL33) tumor cells, obtaining an IC_50_ of 54.78 and 25.23 μg/mL. The study indicated that the autophagy-related proteins microtubule-associated protein 1 (LC3B), sequestration 1 (P62/SQSTM1), autophagy effector protein Beclin1 (Beclin1), and lysosome-associated membrane protein 1 (LAMP1) were positively expressed [38].

Other reports have provided evidence of the antitumor activity of C4 and C9 in several types of human cancer, including breast, lung, colon, prostate, pancreatic, and liver cancer [39,40]. The findings reported for C9 in prostate cancer involve inhibition of the phosphoinositide 3-kinase (PI3K) and protein kinase B (Akt) signaling pathway that ultimately triggers apoptosis [41]. In lung cancer cells (A549), C9 caused cell arrest in the G0/G1 phase and subsequently induced apoptosis. In addition, in lymphoblastic leukemia T (Molt4-hyg), C9 triggered apoptosis via the intrinsic pathway [42].

Subsequently, we perform computer analyses to obtain predictive values using bioinformatics tools. It should be noted that the three compounds with the best activity in vivo and in vitro were used for the analysis, so the terpenoids selected were C3, C9, and C6. In addition, considering that C3 is a mixture of geranial (C3A) and neral (C3B), they were also added to the study. First, we decided to obtain the physical, pharmacokinetic, and toxicological predictive values of terpenoids (Table 3). The results showed that all terpenoids tested could be good candidates for drug development based on the criteria of similarity of physicochemical parameters to other existing drugs described in Lipinski’s rule 5 (Ro5) [43]. In addition, its pharmacokinetic parameters were optimal and acceptable values. However, for plasma protein binding, all terpenoids obtained values > 90%, which could directly influence their oral bioavailability. On the other hand, it would be expected that due to the high lipoficity of terpenoids they could be possible inhibitors of drug-metabolizing enzymes, but most terpenoids did not turn out to be inhibitors or substrates of any of the cytochrome P450 isoenzymes analyzed. However, C8 and C9 may be potential inhibitors of the CYP1A2 isoenzyme, and C3A and C3B were found to be potential inhibitors and substrate of the CYP2C19. Some in vitro studies in human and rat liver microsomes have shown that some acyclic sesquiterpenes, including farnesol, could affect CYP1A-, CYP2B-, and CYP3A-mediated metabolism [44]. All these results could propose terpenoids as molecules that could be used as antitumor agents; however, more research is still needed in in vivo models to confirm the results obtained through predictive values of bioinformatics tools.

Finally, we performed molecular docking studies using terpenoids C3A, C3B, C9, and C6 as ligand, and the targets Bcl-2, HMG-CoA reductase, and FASN were used as targets. Bcl-2 was considered in the study to be of broad importance to NHLs [20] and the numerous studies where terpenoids induce apoptosis in various types of cancer by regulating Bcl-2 [45]. The DHFR enzyme was considered to be the target of the reference drug used in the in vivo and in vitro studies. For the HMG-CoA reductase and FASN targets, terpenoids were selected by analyzing terpenoids in the PASS program and having a high credibility and score; in addition, it was considered that the target was shared for the selected terpenoids (Table 4, Figure 4, Figure 5, Figure 6 and Figure 7). The results for Bcl-2 showed that MTX and C9 showed the best binding energy of −5.8 and −5.76 kcal/mol, respectively. In addition, C9 obtained a polar-like interaction in the Asp 108 residue and all terpenoids shared non-polar-like interactions in Phe 101, Phe 109, Met 112, and Ala 146, important residues for the anti-apoptotic activity reported for Bcl-2 [46,47]. For the DHFR enzyme, MTX obtained the best affinity; however, all terpenoids shared interactions in Ile 7, Leu 22, Arg 31, and Glu 30. These residues have been described as important at the binding site of the hDHFR substrate [48]. On the other hand, the results for FASN showed that C9 obtained the best affinity with a ΔG −5.79 kcal/mol; however, it did not interact with any of the key residues of the enzyme’s active site. What we observed was that all terpenoids interacted with amino acid residues in the interfacial cavity of described importance for the enzyme generated by residues of both subdomains of the enzyme such as Leu 2222, Ile 2250, Phe 2370, and Phe 2371 [49]. Finally, in the results of HMG-CoA reductase, all terpenoids evaluated showed the best affinity for this target and interacted in the Asp 767 residue, a key residue of the enzyme’s catalytic activity [50]. In addition, C9 obtained the best binding energy with a ΔG −6.72 kcal/mol. In general, the theoretical results on the affinity obtained from molecular coupling showed that C9 obtained the best binding energy in the four targets used compared to C3A, C3B, and C6, from which we infer that OH in C1 is relevant for anti-lymphoma activity. Some studies could support our results, as the regulation of acyclic terpenoids on HMG-CoA reductase and its impact on several types of cancer by dysregulating the prenylatation of RAS, Rho, and GTPase proteins that play an important role in cell proliferation and growth processes have been described [29,51,52].

However, further experiments should be carried out to try to elucidate the mechanisms by which the terpenoids C3, C4, C6, and C9 induce anti-lymphoma activity. Finally, it is worth mentioning that we are conducting other relevant experiments on other targets to find findings on the mechanism of action of terpenoids. It is important to note that molecular docking studies, while providing crucial information for drug development, also have several limitations, as they do not reflect the full complexity of the protein microenvironment, the rigid protein, the accuracy of the algorithm, and the water molecules.

## 4. Materials and Methods

### 4.1. Chemicals

Methotrexate, dimethyl sulfoxide (DMSO), L-glutamine, penicillin/streptomycin, and RMPI 1640 medium were purchased from Sigma-Aldrich, St. Louis, MO, USA. Bovine fetal serum was purchased from Gibco, New York, NY, USA. Geranyl acetate (≥97%, *trans*-3,7-Dimethyl-2,6-octadien-1-yl acetate, product number PN: 173495-25G), geranic acid (85%, isomer mixture, 3,7-Dimethyl-2,6-octadienoic acid, PN: 427764-25ML), citral (95%, Lemonal, 3,7-Dimethyl-2,6-octadienal, geranial and neral mixture, PN: C83007-100 ML), geraniol (98%, *E*-3,7-Dimethyl-2,6-octadien-1-ol, PN: 163333-25G), methyl geranate (94%, methyl (2E)-3,7-dimethylocta-2,6-dienoate, isomer mixture, PN: CDS001198), nerol (97%, *Z*-3,7-Dimethyl-2,6-octadien-1-ol, PN: 268909-5ML), citronellicc acid (98%, 3,7-Dimethyl-6-octenoic acid, racemic mix, PN: 303429-25ML), farnesal (≥85%, 3,7,11-Trimethyl-2,6,10-dodecatrienal, isomer mixture, PN: 46188-1ML-F), farnesol (95%, 3,7,11-Trimethyl-2,6,10-dodecatrien-1-ol, isomer mixture, PN: F203-25G), squalene (≥98%, 2,6,10,15,19,23-Hexamethyl-2,6,10,14,18,22-tetracosahexaene, PN: S3626-10ML) were purchased from Sigma-Aldrich^®^ (St. Louis, MO, USA).

### 4.2. Cell-Based Assay

#### 4.2.1. Cell Line

The cell line U-937 (histiocytic lymphoma), diffuse monocytic non-Hodgkin lymphoma was used. The U-937 cell line (ATCC: CRL 1593.2, Middlesex, LDN, UK) was acquired. RPMI 1640 (Roswell Park Memorial Institute) medium supplemented with 2 mM L-glutamine, 10% *v*/*v* of fetal bovine serum, 100 mM of 1% sodium pyruvate and 1% of penicillin/streptomycin was used for cell maintenance and propagation. For all experiments, the cells were kept in an atmosphere with a carbon dioxide (CO_2_) concentration of 5% at 37 °C.

#### 4.2.2. Cytotoxic Activity

Cytotoxic activity against the U-937 cell line was performed using the cell proliferation reagent WST-1, based on the cleavage of tetrazolium salts formed by the mitochondrial enzyme dehydrogenase [53]. The Rapid Cell Proliferation II kit (Abcam, Cambridge, UK, Cat. No. AB65475) was used according to the manufacturer’s instructions. Additionally, 96-well plates were used and 2 × 10^5^ cells per well were seeded in a final volume of 100 μL/well of culture medium. Subsequently, the treatments were added to four concentrations and incubated for 24 h. The concentrations were used in a range of 0.03–0.83 mM; these concentrations were adjusted for each terpenoid because in the preliminary tests it was observed that the cytotoxic activity of some terpenoids had a weak effect and for another strong one. Therefore, the concentrations to be evaluated were adjusted according to these data and obtain a CC_50_ more accurately. For controls, the cells were treated with 1% DMSO and only with RPMI 1640 medium. At the end of the incubation period, 10 μL of WST-1 reagent was added and the cells were incubated again for 2 h. Subsequently, absorbance was measured at 440 nm using a microplate reader (Corning; Reynosa, Mexico). The experiments were carried out in triplicate (using one plate for each compound considering that they are volatile molecules) and independently with the optimal and safety conditions indicated in the specifications of the compounds.

### 4.3. Animals

The experimental animals were provided by the IMSS, using 8-week-old adult male mice of the Balb/c strain and with a body weight of 20 ± 5 g. The procedures and the experimentation protocol were approved by the Bioethics Committee of the Specialty Hospital of the National Medical Center “Siglo XXI”. The registration numbers corresponded to R-2020-3601-186 and R-2019-3601-004. In addition, the study was carried out under the guidelines of the official Mexican standard NOM 0062-ZOO-1999 entitled “Technical Specifications for the Production, Care and Use of Laboratory Animals” [54]. During the biological tests, the animals were kept in controlled conditions with a light/dark cycle of 12 h at 22 °C ± 2 °C. Food and water were available ad libitum.

### 4.4. Anti-lymphoma Activity

To determine anti-lymphoma activity, lymphoma was induced following the procedure described by Calzada et al. [55]. The first step was to form thirteen groups of male mice (*n* = 6). Afterwards, cell inoculation was carried out, for which 1 × 10^6^ U-937 cells were administered intraperitoneally; the animals were kept under observation for the next 28 days. On day 29, the corresponding treatments were administered for each group, the terpenoids geranyl acetate (C1), geranic acid (C2), citral (C3), geraniol (C4), methyl geranate (C5), nerol (C6), citronellic acid (C7), farnesal (C8), farnesol (C9), and squalene (C10) were administered at the dose of 10 mg/kg body weight. The reference drug MTX was administered at a dose of 1.25 mg/kg body weight. The groups healthy control (untreated), the healthy control group (vehicle, tween 80, 2% *v*/*v* in water), the negative control without treatment (U-937). The treatments were administered orally for 9 days and were only performed once a day. After the administration of the treatments, the experimental animals were kept under observation for the next 28 days. During all experiments, the animals were kept under the same conditions of humidity and temperature, and a record of their body weight, condition, and behavior was kept.

Finally, on day 65, the left and right axillary and inguinal lymph nodes were removed and weighed. Anti-lymphoma activity was determined by comparing the total lymph node weight of each group with the control groups and the U-937 group.

### 4.5. In Silico Toxicology and Pharmaceutical Properties

To obtain the pharmacokinetic, toxicological, and physicochemical properties of the acyclic terpenoids that obtained the best inhibitory activity of ganglion growth, the following computer tools were used: Molinspiration [56], SwissADME [57], ADMETlab [58], and Tox-prediction [59]. These predictors are based on the measurement of the pharmacological nature and chemical compatibility with medicine of one or more small molecules and support the possible recognition of candidate molecules in the development of antitumor agents.

### 4.6. Studies of Molecular Docking of Acyclic Terpenoids

To perform molecular docking, acyclic terpenoids obtained from the pubchem database (https://pubchem.ncbi.nlm.nih.gov/, accessed on 12 January 2025), geranial (CID: 638011), neral (CID: 643779), nerol (CID: 643820), farnesol (CID: 3327), navitoclax (CID: 24978538), methotrexate (CID: 126941), HMG-CoA reductase substrate (CID: 5288565), and Orlistad (CID: 14607536). The ligands were optimized and subjected to energetic and geometric minimization using the Avogadro software [60]. Subsequently, the proteins Bcl-2 (PDB ID: 4LVT), DHFR (PDB ID: 3EIG), HMG-CoA reductase (PDB ID: 1DQ9), and FASN (PDB ID: 2PX6) were downloaded from the Protein Data Bank (RCSB) (http://www.rcsb.org/, accessed on 15 January 2025). The enzyme was then prepared to maintain the conditions required in the study. To do this, the total water molecules that they did not need for their catalytic activity were extracted. What was not extracted was the zinc ion, as it is important for its catalytic activity. All polar hydrogen atoms, ionized in a basic environment (pH = 7.4), were aggregated and assigned Gasteiger charges, and the output topologies calculated from the previous steps were used as input files for the coupling simulations.

Theoretical molecular coupling experiments were carried out using Autodock 4.2 software [61]. A grid box of 90 × 90 × 90 Å was used at each spatial coordinate, with a grid point spacing of 0.375 Å. The Lamarckian genetic algorithm was used as a scoring function with a random initial population of 100 individuals and a maximum number of energy assessments of 1 × 10^7^ cycles. The coupled complexes were visualized in the Discovery Studio (BIOVIA, Dassault Systèmes, Discovery Studio Visualizer, 4.5, Dassault Systèmes, San Diego, CA, USA) and PyMOL (The PyMOL Molecular Graphics System, Ver 2.0, Schrödinger, LLC, DeLano Scientific, San Carlos, CA, USA). The validation of the molecular coupling was carried out by recoupling the cocrystallized ligand for each of the targets. The RMSD was calculated and considered a reliable range within 2.2 Å; the calculation was performed by superimposing the cocrystallized ligand with the lowest energy and it was observed if it maintained the same binding position.

### 4.7. Statistical Analysis

The results were expressed as mean ± standard error of six measurements. The results were analyzed using the GraphPad Prisma version 8.4 program (GraphPad software, San Diego, CA, USA). The one-way ANOVA was used, as well as Dunnet’s multiple comparison tests with a value of *p* ˂ 0.05 to establish that there were significant differences between the study groups. The CC_50_ was obtained by linear regression analysis.

## 5. Conclusions

Analysis of the in vitro and in vivo results suggests that the terpenoids citral, geraniol, nerol, and farnesol could be potential anti-lymphoma agents associated with U-937 cells. In addition, structure–activity analysis indicated that inhibition of lymph node growth could be due to the presence of the aldehyde, hydroxyl, and acetate functional groups in the C1 of the monoterpene and sesquiterpene structure. In addition, in the bioinformatic analysis on the pharmacokinetic and toxicological properties, terpenoids obtained optimal and accepted values. Regarding molecular docking studies, theoretical results showed that the anti-lymphomatic activity of terpenoids C3A, C3B, C6, and C9 could be correlated by inhibition of the enzyme HMG-CoA reductase. However, given the limitations of theoretical testing with computational tools, more evidence and data would be needed to elucidate the mechanism of action and safety of acyclic terpenoids.

## Figures and Tables

**Table 1:** Median cytotoxic concentration calculated after 24 h of exposure against U-937 cells from acyclic terpenoids and methotrexate (MTX).

Sample	CC_50_ (mM) ^a^
Geranyl acetate (C1)	0.55 ± 0.01 *
Geranic acid (C2)	0.33 ± 0.01 *
Citral (C3)	0.16 ± 0.01 *
Geraniol (C4)	0.17 ± 0.01 *
Methyl geranate (C5)	0.38 ± 0.01 *
Nerol (C6)	0.11 ± 0.01 *
Citronellic acid (C7)	0.34 ± 0.01 *
Farnesal (C8)	0.36 ± 0.01 *
Farnesol (C9)	0.09 ± 0.01 *
Squalene (C10)	0.26 ± 0.01
MTX	0.25 ± 0.01

^a^ CC_50_: Median cytotoxic concentration causing 50% cell death. Calculated by linear regression analysis of percentage mortality against concentration. Data are expressed as mean ± SEM, (*n* = 3). * *p* < 0.05 vs. MTX.

**Table 2:** Percentage of growth inhibition of axillary and inguinal lymph nodes of mice treated with acyclic terpenoids.

Treatment	Inhibition of Lymph Nodes Growth (%)
Geranyl acetate (C1)	65.93 ± 5.27
Geranic acid (C2)	34.68 ± 5.51 *
Citral (C3)	88.06 ± 3.44 *
Geraniol (C4)	84.28 ± 2.27 *
Methyl geranate (C5)	32.15 ± 4.23 *
Nerol (C6)	75.20 ± 4.33 *
Citronellic acid (C7)	40.16 ± 2.58
Farnesal (C8)	43.82 ± 3.75
Farnesol (C9)	71.37 ± 3.86 *
Squalene (C10)	14.42 ± 1.96 *
MTX	56.17 ± 3.11

Data are expressed as mean ± SEM, (*n* = 6). * *p* < 0.05 vs. MTX.

**Table 3:** Physicochemical, pharmacokinetic, and toxicologic predictive values of acyclic terpenoids ^a^.

Acyclic Terpenoid	C3A	C3B	C9	C6	C4
Physicochemical					
TPSA	17.07	17.07	20.23	20.23	20.23
Lipophilicity (logP)	3.28	3.24	4.70	2.51	2.95
Water solubility (logS)	−2.96	−3	−4.41	−2.62	−3.03
Rotatable bonds	4	4	7	4	4
Number of H-bond donors	0	0	1	1	1
Number of H-bond acceptors	1	1	1	1	1
Druglikeness					
Lipinski	Yes	Yes	Yes	Yes	Yes
Ghose	No	No	Yes	No	No
Veber	Yes	Yes	Yes	Yes	Yes
Egan	Yes	Yes	Yes	Yes	Yes
Pharmacokinetic					
Human intestinal absorption	High	High	High	High	High
BBBp	Yes	Yes	Yes	Yes	Yes
Volume of distribution	0.54	0.64	2.20	2.29	1.98
Plasma protein binding	92.5%	93.3%	97.06%	91.09%	90.82%
CYP1A2 inhibitor	No	No	Yes	No	No
CYP2C19 inhibitor	Yes	Yes	No	No	No
CYP2C19 substrate	Yes	Yes	No	No	No
CYP2C9 inhibitor	No	No	No	No	No
CYP2D6 inhibitor	No	No	No	No	No
CYP2D6 substrate	No	No	No	No	No
CYP3A4 inhibitor	No	No	No	No	No
Clearance	7.51	7.51	8.36	9.94	9.77
T1/2	1.69	1.78	0.670	0.903	0.82
Toxicity					
Mutagenic	No	No	No	No	No
Carcinogenic	No	No	No	No	No
Neurotoxicity	No	-	No	No	No
Rat Oral Acute Toxicity	No	Low	No	No	No
H-HT	Low	Low	Low	Low	Low
Predicted Toxicity Class ^b^	5	4	5	5	5

Topological polar surface area (TPSA), logarithm of the n-octanol/water distribution coefficient (logP), blood–brain barrier permeability (BBBp), human hepatotoxicity (H-HT). ^a^ Predictions were based on SwissADME, ADMETlab, and Tox-prediction web servers, and they were ^b^ Class I: fatal if swallowed (LD_50_ ≤ 5), Class II: fatal if swallowed (5 < LD_50_ ≤ 50), Class III: toxic if swallowed (50 < LD_50_ ≤ 300), Class IV: harmful if swallowed (300 < LD_50_ ≤ 2000), Class V: may be harmful if ingested (2000 < LD_50_ ≤ 5000), Class VI: non-toxic (LD_50_ > 5000).

**Table 4:** Interactions of acyclic terpenoids with amino acid residues at the binding sites of Bcl-2, DHFR, HMG-CoA reductase, and FASN.

Compound	Bcl-2		
	ΔG (kcal/mol)	H-BR	NPI
C3A	−5.07	Ser 113, Glu 133, Phe 147, Glu 149	Phe 101, Phe 109, Met 112, Val 130, Leu 134, Ala 146, Phe 150, Val 153
C3B	−4.83	Ser 113, Val 131, Phe 147, Glu 149, Phe 150	Phe 101, Phe 109, Met 112, Val 130, Leu 134, Ala 146, Val 153
C9	−5.76	Asp 108, Ser 113, Glu 133, Glu 149, Phe 150	Phe 101, Phe 109, Met 112, Val 130, Leu 134, Ala 146, Val 153
C6	−5.17	Val 130, Glu 133, Phe 147, Glu 149	Phe 101, Phe 109, Met 112, Leu 134, Ala 146, Phe 150, Val 153
MTX	−5.8	Ala 97, Asp 100, Phe 101, Arg 104, Tyr 105, Gly 142, Arg 143, Ala 146, Leu 198, Tyr 199, Gly 200	Val 145
Compound	DHFR		
C3A	−4.51	Val 8, Leu 22, Trp 24, Pro 26, Leu 27, Glu 30, Phe 134, Thr 136, Phe 179	Ile 7, Ala 9, Arg 31, Tyr 33, Phe 34
C3B	−4.48	Val 8, Leu 22, Trp 24, Pro 26, Leu 27, Glu 30, Thr 136, Phe 179	Ile 7, Ala 9, Arg 31, Tyr 33, Phe 34
C9	−5.61	Ala 9, Val 10, Ile 16, Trp 24, Pro 26, Leu 27, Glu 30, Thr 56, Val 115, Trp 121, Thr 136	Ile 7, Val 8, Leu 22, Arg 31, Tyr 33, Phe 34
C6	−4.82	Val 8, Ala 9, Val 10, Ile 16, Leu 22, Trp 24, Glu 30, Thr 56, Val 115, Tyr 121, Thr 136	Ile 7, Tyr 33, Phe 34
MTX	−8.58	Ile 7, Val 8, Glu 30, Arg 31, Tyr 33, Phe 34, Thr 56, Ser 59, Pro 61, Asn 64, Leu 67, Val 115, Tyr 121, Thr 136, Phe 179	Ala 9, Leu 22, Ile 60
Compound	HMG-CoA reductase		
C3A	−5.52	Gly 656, Met 657, Gly 765, Asp 767, Glu 801, Ile 802, Gly 803, Val 805, Gly 806, Gly 807, Thr 809	Ala 654, Met 655
C3B	−5.5	Gly 656, Met 657, Gly 765, Gln 766, Asp 767, Glu 801, Ile 802, Gly 803, Val 805, Gly 806, Gly 807, Gly 808, Thr 809	Ala 654
C9	−6.72	Gly 656, Asn 658, Lys 691, Gly 765, Gln 766, Asp 767, Gln 770, Glu 801, Ile 802, Gly 803, Val 805, Gly 806, Gly 807, Thr 809	Ala 654, Met 655, Met 657, Met 659
C6	−5.87	Gly 656, Met 657, Gly 765, Asp 767, Gln 770, Glu 801, Ile 802, Gly 803, Val 805, Gly 806, Gly 807, Gly 808, Thr 809	Ala 654
MTX	−5.26	Tyr 517, Val 522, Cys 526, Tyr 533, Met 534, Pro 535, Ile 536, Pro 537, Val 538, Thr 557, Thr 558, Tyr 761, Ile 762, Ala 763, Cys 764, Gly 765 Gln 766, Ala 768, Gly 808, Leu 811, Pro 813	Pro 535
Compound	FASN		
C3A	−4.86	Ile 2250, Glu 2251, Gln 2374, Ser 2422, Lys 2426	Leu 2222, Leu 2223, Phe 2370, Phe 2371, Phe 2375. Ala 2419, Phe 2423
C3B	−4.8	Leu 2222, Ile 2250, Glu 2251, Gln 2374, Ser 2422, Lys 2426	Leu 2223, Phe 2370, Phe 2371, Phe 2375, Ala 2419, Phe 2423
C9	−5.79	Leu 2223, Pro 2249, Glu 2251, Gln 2374, Phe 2375, Ser 2422, Lys 2426	Ala 2419, Leu 2222, Ile 2250, Phe 2370, Phe 2371, Phe 2423
C6	−5.09	Ile 2250, Glu 2251, Gln 2374, Ala 2419, Ser 2422, Lys 2426	Leu 2222, Leu 2223, Val 2224, Phe 2370, Phe 2371, Phe 2375, Phe 2423
MTX	−4.93	Leu 2222, Leu 2223, Ile 2250, Glu 2251, Val 2256, Phe 2370, Phe 2371, Gln 2374, Phe 2375, Ala 2419, Phe 2423, Arg 2482	-

ΔG: Binding energy (kcal/mol); H-BR: H-binding residues; NPI: Non-polar interactions; Ala: Alanine; Asp: Aspartate; Asn: Asparagine; Arg: Arginine; Cys: Cysteine; Lys: Lysine; Thr: Threonine; Met: Methionine; Ser: Serine; Trp: Tryptophan; Leu: Leucine; Gly: Glycine; Glu: Glutamic acid; Ile: Isoleucine; Tyr: Tyrosine; Pro: proline; Phe: phenylalanine; Gln: Glutamine; Val: Valine. All results were obtained through docking approaches.

## Data Availability

The data presented or additional data in this study are available on request from the corresponding author.

## References

[1] Sun L., Romancik J.T. (2025). The Development and Application of Bispecific Antibodies in B-Cell Non-Hodgkin Lymphoma. J. Pers. Med..

[2] Sapkota S., Shaikh H. (2023). Non-Hodgkin Lymphoma.

[3] Luo J., Craver A., Bahl K., Stepniak L., Moore K., King J., Aschebrook-Kilfoy B. (2022). Etiology of non-Hodgkin lymphoma: A review from epidemiologic studies. J. Natl. Cancer Cent..

[4] Secretaria de Salud Publica. https://www.gob.mx/salud/prensa/294-mexico-registra-al-ano-mas-de-195-mil-casos-de-cancer-secretaria-de-salud#:~:text=Del%20linfoma%20no%20Hodgkin%20hay,en%20mayores%20de%2060%20a%C3%B1os.

[5] Leick M.B., Maus M.V., Frigault M.J. (2021). Clinical perspective: Treatment of aggressive B cell lymphomas with FDA-approved CAR-T cell therapies. Mol. Ther..

[6] Matasar M.J., Luminari S., Barr P.M., Barta S.K., Danilov A.V., Hill B.T., Okosun J. (2019). Follicular lymphoma: Recent and emerging therapies, treatment strategies, and remaining unmet needs. Oncologist.

[7] Ansell S.M. (2015). Hodgkin lymphoma: Diagnosis and treatment. Mayo Clin. Proc..

[8] Baena-Gómez M.A., Matilla M.M., Atienza A.L., Catalán M.A., Marqués C.H., López L.M. (2015). Non-Hodgkin lymphoma: Excellent results at the expense of the high toxicity of the treatment. An. Pediatría.

[9] Al-Naeeb A.B., Ajithkumar T., Behan S., Hodson D.J. (2018). Non-hodgkin lymphoma. BMJ.

[10] Wang Q., Zhao X., Jiang Y., Jin B., Wang L. (2023). Functions of representative terpenoids and their biosynthesis mechanisms in medicinal plants. Biomolecules.

[11] Paduch R., Kandefer-Szerszeń M., Trytek M., Fiedurek J. (2007). Terpenos: Sustancias útiles en la salud humana. Arch. Immunol. Ther. Exp..

[12] Masyita A., Sari R.M., Astuti A.D., Yasir B., Rumata N.R., Emran T.B., Simal-Gandara J. (2022). Terpenes and terpenoids as main bioactive compounds of essential oils, their roles in human health and potential application as natural food preservatives. Food Chem. X.

[13] Kamran S., Sinniah A., Abdulghani M.A., Alshawsh M.A. (2022). Therapeutic potential of certain terpenoids as anticancer agents: A scoping review. Cancers.

[14] NCT02255084. Vaginal Self-Sampling and Human Papillomavirus Testing in Unscreened Women. NCT02255084.

[15] Cortellini A., Verna L., Cannita K., Napoleoni L., Parisi A., Ficorella C., Porzio G. (2017). Topical menthol for treatment of chemotherapy-induced peripheral neuropathy. Indian J. Palliat. Care.

[16] Mu J., Gao S., Mao Y., Xue Q., Yuan Z., Li N., Su K., Yang K., Lv F., Qiu B. (2015). Open three-stage transthoracic oesophagectomy versus minimally invasive thoraco-laparoscopic oesophagectomy for oesophageal cancer: Protocol for a multicentre prospective, open and parallel, randomised controlled trial. BMJ Open.

[17] Miller J.A., Thomson P., Hakim I.A., Lopez A.M., Vining D., Chew W.M., Chow H.H.S. (2010). Abstract A79: Human breast tissue bioavailability of topically applied limonene. Can Prev Res..

[18] Passiglia F., Listì A., Castiglia M., Perez A., Rizzo S., Bazan V., Russo A. (2017). EGFR inhibition in NSCLC: New findings and opened questions?. Crit. Rev. Oncol. Hematol..

[19] Davids M.S., Roberts A.W., Seymour J.F., Pagel J.M., Kahl B.S., Wierda W.G., Gerecitano J.F. (2017). Phase I first-in-human study of venetoclax in patients with relapsed or refractory non-Hodgkin lymphoma. J. Clin. Oncol..

[20] Cheah C.Y., Fowler N.H., Wang M.L. (2016). Breakthrough therapies in B-cell non-Hodgkin lymphoma. Ann. Oncol..

[21] Fowler-Shorten D.J., Hellmich C., Markham M., Bowles K.M., Rushworth S.A. (2024). BCL-2 inhibition in haematological malignancies: Clinical application and complications. Blood Rev..

[22] Rana R.M., Rampogu S., Zeb A., Son M., Park C., Lee G., Lee K.W. (2019). In silico study probes potential inhibitors of human dihydrofolate reductase for cancer therapeutics. J. Clin. Med..

[23] Rao A.S., Tapale S.R. (2013). A Study on dihydrofolate reductase and its inhibitors: A review. Int. J. Pharma Sci. Res..

[24] Peeters R., Cuenca-Escalona J., Zaal E.A., Hoekstra A.T., Balvert A.C., Vidal-Manrique M., Van Spriel A.B. (2022). Fatty acid metabolism in aggressive B-cell lymphoma is inhibited by tetraspanin CD37. Nat. Commun..

[25] Cuyàs E., Pedarra S., Verdura S., Pardo M.A., Espin Garcia R., Serrano-Hervás E., Menendez J.A. (2024). Fatty acid synthase (FASN) is a tumor-cell-intrinsic metabolic checkpoint restricting T-cell immunity. Cell Death Discov..

[26] Schroeder B., Vander Steen T., Espinoza I., Venkatapoorna C.M.K., Hu Z., Silva F.M., Lupu R. (2021). Fatty acid synthase (FASN) regulates the mitochondrial priming of cancer cells. Cell Death Discov..

[27] Juarez D., Fruman D.A. (2021). Targeting the mevalonate pathway in cancer. Trends Cancer.

[28] Mullen P.J., Yu R., Longo J., Archer M.C., Penn L.Z. (2016). The interplay between cell signalling and the mevalonate pathway in cancer. Nat. Rev. Cancer.

[29] Yeganehjoo H., DeBose-Boyd R., McFarlin B., Mo H. (2017). Synergistic impact of d-δ-tocotrienol and geranylgeraniol on the growth and HMG CoA reductase of human DU145 prostate carcinoma cells. Nutr. Cancer.

[30] Ramírez-Santos J., Calzada F., Ordoñez-Razo R.M., Mendieta-Wejebe J.E., Velázquez-Domínguez J.A., Argüello-García R., Barbosa E. (2024). In vivo, in vitro and in silico anticancer activity of ilama leaves: An edible and medicinal plant in México. Molecules.

[31] Ramírez-Santos J., Calzada F., Mendieta-Wejebe J.E., Ordoñez-Razo R.M., Martinez-Casares R.M., Valdes M. (2022). Understanding the anti-lymphoma activity of Annona macroprophyllata Donn and its acyclic terpenoids: In vivo, in vitro, and in silico studies. Molecules.

[32] Koźmiński P., Halik P.K., Chesori R., Gniazdowska E. (2020). Overview of dual-acting drug methotrexate in different neurological diseases, autoimmune pathologies and cancers. Int. J. Mol. Sci..

[33] Abramson J.S., Palomba M.L., Gordon L.I., Lunning M.A., Wang M., Arnason J., Siddiqi T. (2020). Lisocabtagene maraleucel for patients with relapsed or refractory large B-cell lymphomas (TRANSCEND NHL 001): A multicentre seamless design study. The Lancet.

[34] Zubair A.C., Ali S.A., Rees R.C., Goepel J.R., Winfield D.A., Goyns M.H. (1996). Analysis of the colonization of unirradiated and irradiated SCID mice by human lymphoma and non-malignant lymphoid cells. Leuk. Lymphoma.

[35] Qi F., Yan Q., Zheng Z., Liu J., Chen Y., Zhang G.J. (2018). Geraniol and geranyl acetate induce potent anticancer effects in colon cancer Colo-205 cells by inducing apoptosis, DNA damage and cell cycle arrest. J. BUON.

[36] Saini D., Chaudhary P.K., Chaudhary J.K., Kaur H., Verma G.K., Pramanik S.D., Prasad R. (2024). Molecular mechanisms of antiproliferative and pro-apoptotic effects of essential oil active constituents in MCF7 and T24 cancer cell lines: In vitro insights and in silico modelling of proapoptotic gene product-compound interactions. Apoptosis.

[37] Silva G.D.S.E., Marques J.N.J., Linhares E.P.M., Bonora C.M., Costa É.T., Saraiva M.F. (2022). Review of anticancer activity of monoterpenoids: Geraniol, nerol, geranial and neral. Chem. Biol. Interact..

[38] Chen L., Zhang Y.J., Qin H.W., Hu W.J., Liu Y.J., Xiao G.S., Yang D.L. (2024). Study on mechanism of inhibiting proliferation of head and neck cancer cells by citral, active ingredient of lemon essential oil. J. Chin. Mater. Med..

[39] Cho M., So I., Chun J.N., Jeon J.H. (2016). The antitumor effects of geraniol: Modulation of cancer hallmark pathways. Int. J. Oncol..

[40] Jung Y.Y., Hwang S.T., Sethi G., Fan L., Arfuso F., Ahn K.S. (2018). Potential anti-inflammatory and anti-cancer properties of farnesol. Molecules.

[41] Carver B.S., Chapinski C., Wongvipat J., Hieronymus H., Chen Y., Chandarlapaty S., Arora V.K., Le C., Koutcher J., Scher H. (2011). Reciprocal feedback regulation of PI3K and androgen receptor signaling in PTEN-deficient prostate cancer. Cancer Cell.

[42] Joo J.H., Ueda E., Bortner C.D., Yang X.P., Liao G., Jetten A.M. (2015). Farnesol activates the intrinsic apoptosis pathway and the ATF4-ATF3-CHOP cascade of ER stress in human T-lymphoblastic leukemia Molt4 cells. Biochem. Pharmacol..

[43] Pollastri M.P. (2010). Overview on the Rule of Five. Curr. Protoc. Pharmacol..

[44] Špičáková A., Szotáková B., Dimunová D., Myslivečková Z., Kubíček V., Ambrož M., Skálová L. (2017). Nerolidol and farnesol inhibit some cytochrome P450 activities but did not affect other xenobiotic-metabolizing enzymes in rat and human hepatic subcellular fractions. Molecules.

[45] Guo J., Huang M., Hou S., Yuan J., Chang X., Gao S., Li J. (2024). Therapeutic Potential of Terpenoids in Cancer Treatment: Targeting Mitochondrial Pathways. Cancer Rep..

[46] Moradipoodeh B., Jamalan M., Zeinali M., Fereidoonnezhad M., Mohammadzadeh G. (2019). Actividad anticancerígena in vitro e in silico de la amigdalina en la línea celular de cáncer de mama humano SK-BR-3. Mol. Biol. Rep..

[47] Souers A.J., Leverson J.D., Boghaert E.R., Ackler S.L., Catron N.D., Chen J., Elmore S.W. (2013). ABT-199, a potent and selective BCL-2 inhibitor, achieves antitumor activity while sparing platelets. Nat. Med..

[48] Rana R.M., Rampogu S., Abid N.B., Zeb A., Parate S., Lee G., Lee K.W. (2020). In silico study identified methotrexate analog as potential inhibitor of drug resistant human dihydrofolate reductase for cancer therapeutics. Molecules.

[49] Pemble C.W., Johnson L.C., Kridel S.J., Lowther W.T. (2007). Crystal structure of the thioesterase domain of human fatty acid synthase inhibited by Orlistat. Nat. Struct. Mol. Biol..

[50] Istvan E.S., Deisenhofer J. (2001). Structural mechanism for statin inhibition of HMG-CoA reductase. Science.

[51] Mo H., Jeter R., Bachmann A., Yount S.T., Shen C.L., Yeganehjoo H. (2019). The potential of isoprenoids in adjuvant cancer therapy to reduce adverse effects of statins. Front. Pharmacol.

[52] Galle M., Crespo R., Rodenak Kladniew B., Montero Villegas S., Polo M., de Bravo M.G. (2014). Suppression by geraniol of the growth of A549 human lung adenocarcinoma cells and inhibition of the mevalonate pathway in culture and in vivo: Potential use in cancer chemotherapy. Nutr. Cancer..

[53] Joo K.M., Kim S., Koo Y.J., Lee M., Lee S.H., Choi D., Lim K.M. (2019). Development and validation of UPLC method for WST-1 cell viability assay and its application to MCTT HCE™ eye irritation test for colorful substances. Toxicol. Vitro.

[54] Norma Oficial Mexicana (1999). NOM-062-ZOO-1999: Especificaciones Técnicas Para la Producción, Cuidado y Uso de Los Animales de Laboratorio. https://www.fmvz.unam.mx/fmvz/principal/archivos/062ZOO.PDF.

[55] Calzada F., Solares-Pascasio J.I., Valdes M., Garcia-Hernandez N., Velazquez C., Ordoñez-Razo R.M., Barbosa E. (2018). Anti-lymphoma potential of the ethanol extract and rutin obtained of the leaves from Schinus molle Linn. Pharmacogn. Res..

[56] Molinspiration Cheminformatics 2017 Calculation of Molecular Properties and Bioactivity Score. http://www.molinspiration.com/services/logp.html.

[57] Daina A., Michielin O., Zoete V. (2017). SwissADME: A free web tool to evaluate pharmacokinetics, drug-likeness and medicinal chemistry friendliness of small molecules. Sci. Rep..

[58] Xiong G., Wu Z., Yi J., Fu L., Yang Z., Hsieh C., Cao D. (2021). ADMETlab 2.0: An integrated online platform for accurate and comprehensive predictions of ADMET properties. Nucleic Acids Res..

[59] Banerjee P., Kemmler E., Dunkel M., Preissner R. (2024). ProTox 3.0: A webserver for the prediction of toxicity of chemicals. Nucleic Acids Res..

[60] Hanwell M., Curtis D., Lonie D., Vandermeersch T., Zurek E., Hutchison G. (2012). Avogadro: An advanced semantic chemical editor, visualization, and analysis platform. J. Cheminformatics.

[61] Morris G., Lindstrom W., Sanner M., Belew R., Goodshell D., Olson A. (2009). Autodock4 and AutodockTools4: Automated docking with selective receptor flexibility. J. Comput. Chem..

